# Graphene Improves the Biocompatibility of Polyacrylamide Hydrogels: 3D Polymeric Scaffolds for Neuronal Growth

**DOI:** 10.1038/s41598-017-11359-x

**Published:** 2017-09-08

**Authors:** Cristina Martín, Sonia Merino, Jose M. González-Domínguez, Rossana Rauti, Laura Ballerini, Maurizio Prato, Ester Vázquez

**Affiliations:** 1 Organic Chemistry area, Faculty of Chemical Science and Technology-IRICA, University of Castilla-La Mancha, Avda. Camilo José Cela 10, 13071 Ciudad Real, Spain; 2 Department of Chemical and Pharmaceutical Sciences, University of Trieste, Piazzale Europa 1, 34127 Trieste, Italy; 3 International School for Advanced Studies (SISSA), via Bonomea 265, 34136 Trieste, Italy; 4 Carbon Nanobiotechnology Laboratory, CIC biomaGUNE, Paseo de Miramón 182, 20009 Donostia-San Sebastián, Spain; 5 Ikerbasque, Basque Foundation for Science, E-48011 Bilbao, Spain

## Abstract

In tissue engineering strategies, the design of scaffolds based on nanostructures is a subject undergoing intense research: nanomaterials may affect the scaffolds properties, including their ability to interact with cells favouring cell growth and improving tissue performance. Hydrogels are synthetic materials widely used to obtain realistic tissue constructs, as they resemble living tissues. Here, different hydrogels with varying content of graphene, are synthesised by *in situ* radical polymerization of acrylamide in aqueous graphene dispersions. Hydrogels are characterised focusing on the contribution of the nanomaterial to the polymer network. Our results suggest that graphene is not a mere embedded nanomaterial within the hydrogels, rather it represents an intrinsic component of these networks, with a specific role in the emergence of these structures. Moreover, a hybrid hydrogel with a graphene concentration of only 0.2 mg mL^−1^ is used to support the growth of cultured brain cells and the development of synaptic activity, in view of exploiting these novel materials to engineer the neural interface of brain devices of the future. The main conclusion of this work is that graphene plays an important role in improving the biocompatibility of polyacrylamide hydrogels, allowing neuronal adhesion.

## Introduction

Adapting the microenvironment of three-dimensional (3D) tissue-scaffolds to regulate neural cell adhesion or tissue growth is essential to design new therapies for neurological applications. In these developments, 3D systems are being highly investigated as they are showing, when compared *in vitro* with two-dimensional (2D) systems, better results in relation to neuronal differentiation^1, 2^, neuronal growth^3^ and improvement of functional organization and synchronization in small neuronal assemblies^4, 5^. In general, 3D scaffolds have been designed to accomplish important features for implantable prostheses: biocompatibility, stability and homogeneity at the microscale, conductivity and controlled mechanical properties. Biocompatibility is in general defined as “the ability of a material to perform with an appropriate host response in a specific application”^6^, although new definitions, based also on cell function, are increasingly proposed^7^.

Recently, emerging carbon-based nanomaterials, with their unique physicochemical properties, have opened up additional and novel opportunities for neuronal tissue engineering developments. Among these, carbon nanotubes have been the most inspiring ones^8, 9^. These nanomaterials represent biocompatible substrates, and they can be functionalised with polymers to modulate neuronal survival and growth^10^. Graphene has also attracted the attention of neuroscientists, demonstrating biocompatibility with neurons in 2D interfaces, being able to preserve the basal physiological level of neuronal activity^11–13^. Very interesting examples are related to 3D porous graphene structures^14^, which have been used as new scaffolds for neural stem cells *in vitro*. For example, Li and co-workers^15^ found that the graphene-foam not only supports cells growth, but also keeps them in an active proliferation state better than 2D graphene films.

Hydrogels - 3D polymer networks that possess high water contents - have been used to obtain more realistic tissue constructs because of their ability to mimic the nature of soft tissues^16–18^. Hydrogels are the best candidates to play the role of extracellular matrix for 3D cell culture^19^, mainly due to their aqueous environment and their porous structure, which make it possible to link and maintain proteins in a functional state^20, 21^. This fact allows improving cell culture development by anchoring, for example, growth factors to the polymer network, which makes these systems even more similar to the *in vivo* condition.

Hydrogels can be obtained from a wide range of materials; however, one of the main disadvantages of this kind of polymers is their low mechanical strength. Over the last years there has been a growing interest in reinforcing polymeric hydrogels with nanosized materials to improve and adequate their poor mechanical consistence. Remarkably, the nanomaterials embedded in a cross-linked polymer network not only make the system stiffer and tougher, but can provide novel features, adding synergistic benefits to the hybrid 3D materials^22, 23^. In this direction, many efforts have been made to fabricate hybrid graphene-based hydrogels with enhanced properties, mainly using graphene oxide (GO)^24, 25^, graphene oxide peroxide (GOP)^26^, or even reduced graphene (rGO) from GO^27, 28^. However, most of these studies do not provide details about the role of the nanomaterial within the network, moreover GO sheets are insulating and its electronic properties differ substantially from those of graphene. As a matter of fact, there are almost no examples in literature of pristine graphene-based hydrogels^29^, which are mainly limited due to the difficulties to obtain pristine graphene aqueous solutions in a simple, scalable and cost efficient way.

With the latest advances in graphene research, one can find a handful of recent works that timely address this conceptual approach from top-down cleavage of graphite. Fan *et al*.^30^ chemically modified a polysaccharide with pendant methacrylate groups, which acted as a physical exfoliant of graphite to produce multi-layer graphene flakes, followed by co-polymerization with methacrylic acid and a cross-linker. Toumia and co-workers^31^ dispersed graphite in an aqueous solution of a commercial amphiphilic surfactant containing aromatic moieties (PEG-PEPEMA) which was chemically grafted to a polyvinylalcohol (PVA) hydrogel network. Shim *et al*.^32^ probed the effects of different PVA and dextran polymers containing phenyl or pyrene units for the physical exfoliation of graphite in water and subsequent hydrogelation of the obtained suspensions with glutaraldehyde. Finally, Ahadian and co-workers developed a friendly aqueous route to graphite exfoliation down to few-layer graphene sheets by use of bovine serum albumin in water as exfoliant^33^, and used it to fabricate hydrogel nanocomposites made of methacrylated gelatin.

Here, we present a new class of graphene-based hydrogels and we show their ability to support the growth of living primary neurons. The produced nanocomposites were compared to the hydrogels in the absence of nanomaterials and were fully characterised. The presence of graphene brought about a noticeable improvement in the properties of the hybrid materials and varying the graphene amounts allowed to study its role in the resulting features. Hippocampal neurons and astrocytes developed efficiently only in the graphene-doped hydrogels. In these graphene hybrids, the cultured neurons gave rise to active synaptic networks as observed by immunofluorescence microscopy and Ca^2+^ imaging experiments.

## Methods

### Materials

Graphite powder was acquired from Bay Carbon Inc. (SP-1 reference). Acrylamide (Am), *N*,*N*′-methylenebisacrylamide (MBA), potassium peroxodisulfate (KPS) and melamine were purchased from Sigma Aldrich.

### Preparation of the aqueous graphene dispersions

The exfoliation of graphite was carried out using a ball-milling procedure developed in our group^34^. In a typical experiment, a graphite/melamine mixture (1:3) (30 mg) was ball-milled at 100 rpm for 30 minutes using a Retsch PM100 planetary mill. The resulting solid mixture was dispersed in water (20 mL) to produce a dark suspension, which was then left to settle down for five days while some precipitate (mainly pure or poorly exfoliated graphite) segregated from the liquid. The liquid fraction with stable sheets in suspension was carefully extracted. Melamine was removed by washing the extracted liquid with hot water, leading to few-layer graphene sheets almost without defects. The obtained graphene dispersions were stable at room temperature for some weeks. The zeta potential of the dispersion was measured at 25 °C using Milli-Q water and CRISON buffer solution (pH = 7) by BIC 90Plus analyser.

### Preparation of the pure polyacrylamide hydrogel (AM)

Pure polyacrylamide hydrogels were prepared using Am, MBA as cross-linker and KPS as initiator. Am was initially dissolved in ultrapure Milli-Q water (200 mg mL^−1^) together with the MBA (0.2 mg mL^−1^). The solution was homogenised by stirring and mild sonication. Then, KPS was added (at a final concentration of 0.4 mg mL^−1^) and polymerization took place at 75 °C for 1 hour. The resulting materials were immersed in water for at least four days, and the water was replaced every day in order to remove any unreacted monomer and initiator molecules.

### Preparation of the graphene-based polyacrylamide hydrogels (AMGXs)

A similar process as the previously described for the preparation of AM was followed to synthesise the nanocomposite, adding in this case all the reactants to the aqueous graphene dispersion. Graphene water dispersions with different concentrations were used (between 0.05 mg mL^−1^ and 2 mg mL^−1^).

### Scanning Electron Microscopy (SEM) experiments

The hydrogel microstructure was analysed by SEM, using PHILIPS XL30 and FEI QUANTA 250 systems. The distribution of the pore size was measured for all samples in the various SEM images by using the Fiji® software. Blank and hybrid graphene hydrogels were completely swollen and frozen and then dried overnight in a Telstar Lyoquest freeze-drier to yield dry aerogel samples. SEM samples were prepared by sputter coating with an Au layer of about 15 nm just when they were analysed using the PHILIPS instrument.

### Raman spectroscopy

Raman spectra were recorded with an InVia Renishaw microspectrometer equipped with a 532 nm point-based laser. Power density was kept in all cases below 1 mW/µm^2^ to avoid laser heating effects. The obtained spectra were a result of probing many random locations on each sample. Three-dimensional Raman mappings were performed with the 532 nm laser by using the line-based option (streamline), which analysed 9 × 9 µm^2^ slices of sample and depth steps of 1 µm until reaching 10 µm. According to the dimensions of the probed volume and given that we registered 6200 spectra during the mapping, we end up with a pixel size of 0.13 µm^3^.The whole set of spectra obtained was fitted to the G-band region in three dimensions.

### Mechanical properties

Compressive tests were performed on hydrogels at 25 °C with a Mecmesin Multitest 2.5-i dynamic mechanical analyser. Cylindrical disks of gels (x6) with a diameter of 1.5 cm and an initial thickness of 1.5 cm were molded. Hydrogel disks were uniaxially compressed between two plates at the rate of 6 mm min^−1^ (cell load = 100 N). The extent of the deformation was measured in terms of compression ratio which equals the ratio of the initial height of the sample to the final height of the sample. The extent of the deformation was also measured in terms of strain, which equals the ratio of the displacements of the samples. After holding the constant deformation, the deformed hydrogel was removed from the press. This behaviour was represented in the typical stress-strain curves from which it was possible to obtain the Young’s modulus. In this case that value was calculated between 15% and 20% of strain. Moreover, fatigue behaviour was studied using the aforementioned program in a dynamic way, reaching 100 compressive cycles with each sample. Creep experiments were conducted on hydrogels at ambient conditions with a Mecmesin Multitest 2.5-i dynamic mechanical analyser. Cylindrical disks of gels (×3) with a diameter of 1.5 cm and an initial thickness of 1.5 cm were molded. Hydrogel disks were uniaxially compressed between two plates. The deformed hydrogels were then held under constant applied load at 15% compressional strain for an extended period of time.

### Cell culture

Primary hippocampal cultures were prepared from postnatal (2–3 days) rats as previously reported^35–37^. All procedures were approved by the local veterinary authorities and the SISSA ethical committee, in accordance with the Italian law (decree 26/14) and the UE guidelines (2007/526/CE and 2010/63/EU). The use of animals was approved by the Italian Ministry of Health. All efforts were made to minimise suffering and to reduce the number of animal used. Hippocampi were isolated from the rest of the brain and upon enzymatic treatment and neurons were mechanically dissociated. Cells were plated on three different substrates: poly-L-ornithine-coated, pure polyacrylamide hydrogel (AM) and 0.2 mg mL^−1^ graphene hydrogels (AMG0.2). Before using for culturing, both hydrogels were sliced at 300–400 µm thickness and mounted on the glass coverslip (12 × 24 mm^2^, Kindler, EU) by a thin adhesive layer of Polydimethylsiloxane (PDMS)^5^. One hour prior to plating, hydrogels were treated with an air plasma-cleaner in order to facilitate cell adhesion and at the end sterilised with an UV lamp. Briefly, enzymatically dissociated hippocampal neurons^35, 37^ were suspended in the culture medium consisting of MEM (Gibco), supplemented with glucose (35 mМ), Apo-Transferrin (1 mМ), HEPES (15 mМ), insulin (48 µМ), Biotin (3 µМ), Vitamin B12 (1 mМ) and Gentamicin (Gibco) (500 nМ), Fetal Bovin Serum (FBS, 10%). Cultures were incubated at 37 °C, in a humidified atmosphere with 5% CO_2_ in the same culture medium. Cultures were used for experiments after 8–10 days *in vitro* (DIV).

### Immunofluorescence labelling

After Ca^2+^ measurements, cultures were fixed in 4% formaldehyde (prepared from fresh paraformaldehyde) in PBS for 30 min, at room temperature. Cells were then permeabilised with Triton X-100 (1%) for 45 min, blocked with FBS (5%) in PBS (blocking buffer) for 45 min at room temperature and incubated with primary antibodies for 45 min. The primary antibodies used were: rabbit polyclonal anti-β-tubulin (1:250 dilution) and mouse monoclonal anti-GFAP (1: 500 dilution). After the primary incubation and PBS washes, cultures were incubated for 45 min with Alexa 594 goat anti-rabbit (Invitrogen, 1:500 dilution), Alexa 488 goat anti-mouse (Invitrogen, 1:500 dilution) and with DAPI (Invitrogen, 1:200 dilution) to stain the nuclei. Samples were mounted in Vectashield (Vector Laboratories) on 1 mm thick coverslips and imaged using a confocal microscope (Leica Microsystems GmbH, Wetzlar, Germany). Both 2D-polyornithine and hydrogels substrates were investigated at 20x magnification and serial confocal planes (z-stack) were acquired every 500 nm. The Z profile reconstructions of the images were performed offline using the image-processing package Fiji^38^.

### Calcium imaging

For Ca^2+^ measurements, cultures were loaded with cell permeable Ca^2+^ dye Oregon Green 488 BAPTA-1 AM (Molecular Probes); stock solution of the Ca^2+^ dye (4 mМ) was prepared in DMSO and cultures were incubated with a final concentration of 4 µМ for 30 min at 37 °C in the cell culture incubator. Samples were then placed in a recording chamber mounted on an inverted microscope (Nikon TE-200) where they were continuously superfused by a recording solution of the following composition (in mМ): 150 NaCl, 4 KCl, 2 CaCl_2_, 1 MgCl_2_, 10 HEPES, 10 glucose (pH adjusted to 7.4 with NaOH). The loaded cultures were observed with 40x objective (0.60 NA). Recordings were performed form visual fields (120 × 160 µm^2^) containing on average 6 ± 2 neurons. Prior to recording Ca^2+^ signals, we selected the cells by drawing regions of interest (ROIs) around cell bodies. Images were continuously acquired by a Till Photonics Till-Imago System, exciting the Ca^2+^ dye with a 488 nm wavelength light generated by a monochromator device equipped with integrated light source (Polychrome IV, Till Photonics). Images were acquired continuously for 1800 s (200 ms exposure time) by a cooled slow-scan interline transfer camera (IMAGO CCD camera; Till Photonics) operating on 60 × 80 pixels binning mode. The imaging system was controlled by an integrating imaging software package (TillvisION; Till Photonics) using a personal computer. In order to induce rhythmic bursts, bicuculline methiodide (a blocker of GABA_A_ receptors) (20 µМ) was bath-applied after 10 minutes recording; at the end of each experiments, TTX (1 µМ) (a voltage-gated fast Na^+^ channel blocker; Latoxan) was added to the recording solution to confirm the neuronal nature of the recorded signal. Recorded images were analysed off-line both with the Clampfit software (pClamp suite, 10.2 version; Axon Instrument) and Igor Pro Software (6.32 A version; Wavemetrics, Lake Oswego, Oregon, USA). Intracellular Ca^2+^ signals were expressed as fractional amplitude increase (ΔF/F_0_ where F_0_ is the baseline fluorescence level and ΔF is the rise over baseline); we determined the onset time of neuronal activation by detecting those events in the fluorescent signal that exceed at least five times the standard deviation of the noise. We then computed the difference between consecutive onset times, to obtain the inter-event interval (IEI). All data are presented as mean ± standard deviation (SD) of the mean (n is the number of cells). Statistical significance for IEI was calculated with Student’s t-test (a value of P < 0.05 was accepted as indicative of statistically significant difference; Statistica 6.0 – StatSoft Italy).

### Data availability statement

All data generated or analysed during this study are included in this published article (and its Supplementary Information files).

## Results and Discussion

As the hydrogel formation takes place in aqueous media, the use of nanostructured materials is, in principle, limited to water soluble/dispersible entities. Ideally, the preparation of graphene-containing hydrogels should occur using water suspensions of the nanomaterial in which the hydrogel is going to be formed. For this reason, the use of a stable suspension of graphene sheets in water is crucial. We developed a facile graphite exfoliation method using a ball-milling treatment in the presence of melamine as exfoliating agent, which produces few-layer, non-oxidised graphene. Melamine can be eliminated by washing with hot water^34^. Graphene dispersions in water were obtained and thoroughly characterised (Fig. S1, Supplementary Information). The zeta potential of the aqueous dispersions was 11 ± 4 mV. Elemental analysis of these few-layer graphenes revealed a melamine content of 0.54 wt%^39^.

**Figure 1:**
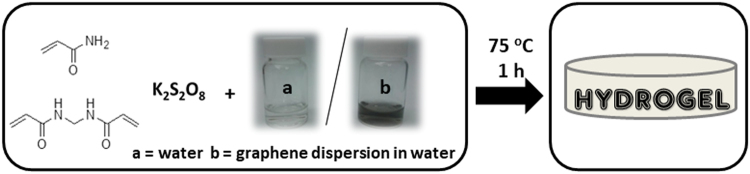
General synthetic scheme for the hydrogels preparation.

Graphene-based hydrogels are formed by *in situ* radical polymerization of acrylamide in water containing graphene suspensions of different concentrations. Potassium peroxodisulfate (KPS) and *N*,*N*′-methylenebisacrylamide (MBA) were chosen as the initiator and the cross-linker respectively. Figure 1 shows the scheme of the process.

**Figure 2:**
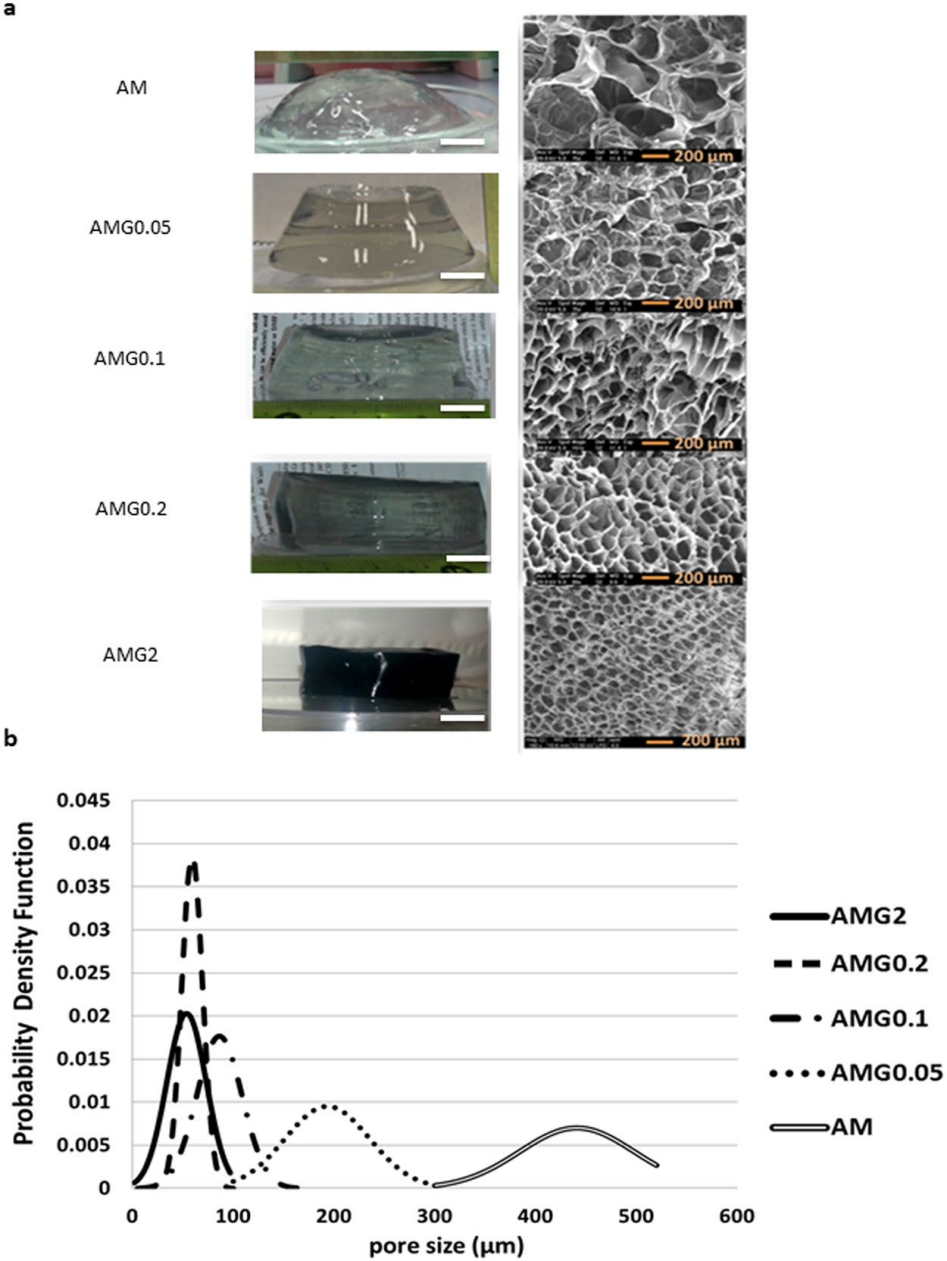
(**a**) Digital (scale bar: 1 cm), SEM pictures (scale bar: 200 µm) and (**b**) pore size distribution of the final fully swollen materials with several graphene concentrations from 0 mg mL^−1^ to 2 mg mL^−1^.

A chemically cross-linked polyacrylamide gel (AM) prepared without graphene and a series of hybrid graphene-polyacrylamide gels (AMGX, being X the graphene concentration used in mg mL^−1^) were synthesised. Figure 2 shows the final aspect of all the materials in the swollen state, which seem completely homogeneous, along with the microscopic morphology of the hydrogels studied by Scanning Electron Microscopy (SEM). The dimensional stability of our hydrogels is quite high, not only in their native swelling state, but also at higher swelling degrees. The higher the graphene content, the better maintenance of the shape with increasing swelling degree.

One important feature for the use of these materials as 3D scaffolds for cell culture is the pore size of the sponge-like structure. In general, the pore size of a hydrogel, in their maximum swelling degree, depends mainly on the amount of cross-linker^40^. In the present case, as shown in Fig. 2, the pore size can be modulated by the amount of graphene present in the structure: the higher is the graphene concentration, the smaller is the pore size of the material. This graphene-induced reduction in pore-size correlates well with the idea that graphene acts as a cross-linker and takes part in the hydrogel structure. This fact also affects, as will be shown later, the swelling behaviour and the mechanical properties of the hybrid material.

**Figure 3:**
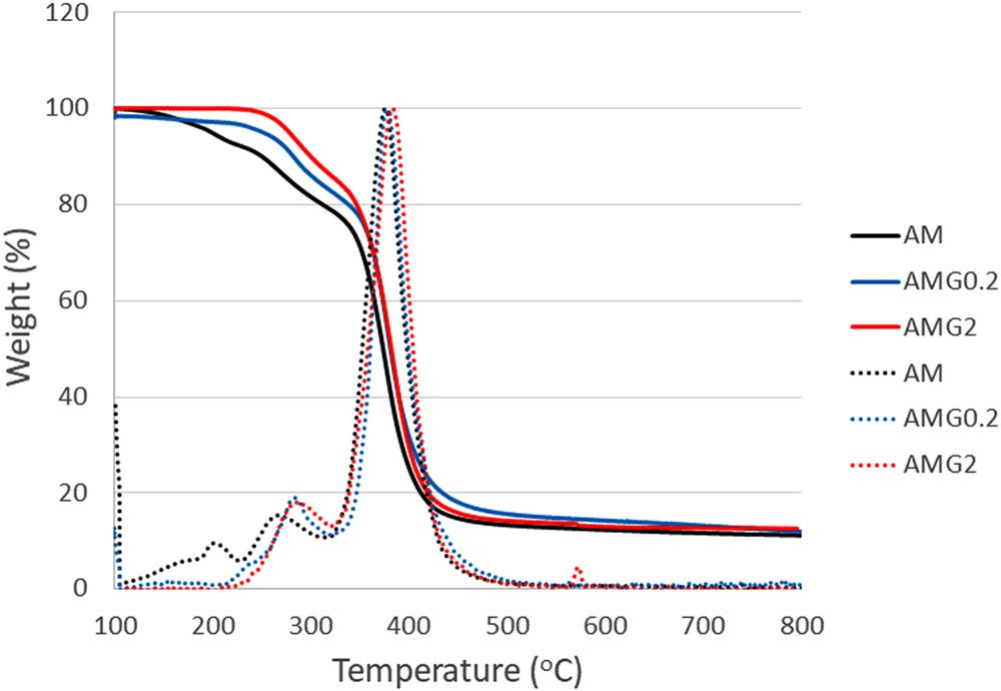
TGA curves (solid line) and derivatives (dotted line) of AM, AMG0.2 and AMG2 hydrogels.

Graphene and crosslinker (MBA) are used in similar proportions. Moreover, we can exclude a relevant role of the residual melamine in the hydrogel structure, because the amount of melamine in the final gels is negligible. Hydrogels can be chemically or physically crosslinked^41, 42^. In the first case, hydrogels are mainly synthesised by chain growth polymerization. Regarding physically crosslinked hydrogels, the polymer networks can be synthesised by ionic interactions, hydrogen bonds, hydrophobic interactions or even crystallisation. Taking into account the physico-chemical characteristics of the graphene material used in these experiments, with low number of defects and very low quantity of oxygen atoms, interactions such as hydrogen bonds, commonly considered for GO^24^ can be excluded. We have further characterised our hydrogels by thermogravimetric analysis (TGA) and the results show structural changes in the hydrogels with graphene compared to the sample without nanomaterial (Fig. 3). Three weight loss regions are observed in AM (Table 1). The first weight loss takes place around 200 °C and corresponds to the NH_3_ loss from the primary amide groups^43^. Small amounts of CO_2_ are released around 267 °C, and the main weight loss occurs at 375 °C, which corresponds to the aliphatic backbone of the polymer matrix. In the case of AMG0.2, the NH_3_ loss shifts to higher temperatures and almost disappears, presumably due to a strong interaction of the nitrogen of the amide group and the graphene surface. In fact, the first weight loss is not observed in the hydrogel with the highest graphene concentration, AMG2. Moreover, under the conditions used in our polymerization reaction, we generate radicals that can obviously attack the graphene extended π-system. In fact, it is known that graphene can be used as a crosslinker in polymerisation processes, in order to produce novel functional materials^44, 45^.

**Table 1:** Characteristic temperatures for TGAs of AM, AMG0.2 and AMG2 hydrogels.

Temperature (°C)			
Sample	Peak 1	Peak 2	Peak 3
AM	199	267	375
AMG0.2	240	282	376
AMG2	—	285	383

**Figure 4:**
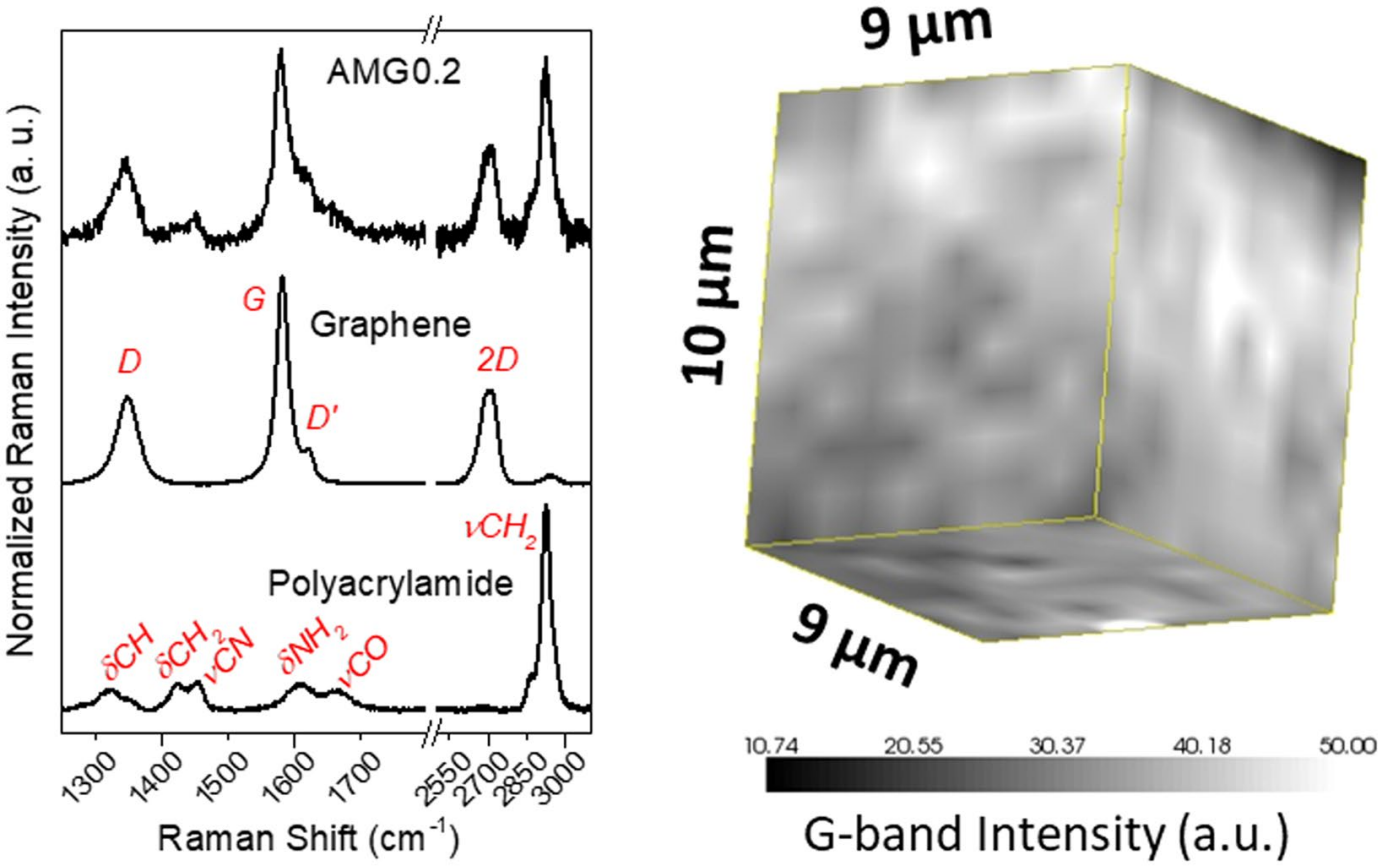
(left) Representative Raman spectra (532 nm laser) of MBA-crosslinked polyacrylamide matrix (AM), graphene and the resulting nanocomposite; (right) 3D map of the *G*-band intensity in AMG0.2.

Raman spectroscopy was also used to identify the presence of graphene within the hydrogel and to study its distribution in the polymer matrix. As can be observed in Fig. 4 (left), the neat hydrogel matrix presents Raman bands that, according to the literature^46^, are ascribed to the different stretching (*ν*) and bending (δ) vibrations of the polyacrylamide skeleton, being the most intense the methylene and *C-N* stretchings, and bending of amino groups. Upon acquisition of Raman spectra from the hybrid graphene hydrogels, the characteristic features of graphene are visible, mainly the disordered carbon (*D*-band), the sp^2^ tangential mode (*G*-band), and the second-order vibration mode (*2D*-band). Raman spectroscopy was also employed to reveal the state of dispersion of graphene within the hydrogel matrix, throughout three-dimensional mappings at a micrometer scale (Fig. 4 (right)). A sample volume of 9 × 9 × 10 µm^3^ was probed, and the intensity of the *G*-band was mapped across such a volume. A fair distribution of graphene is noticed with no apparent aggregates at the micron scale, supporting the success of our water dispersion approach to obtain homogeneous hydrogel nanocomposites.

### Swelling behaviour

The swelling ratio is an important parameter since it describes the amount of water that is contained within the hydrogel. It is a function of the network structure, hydrophilicity, and degree of ionization of the functional groups, but mainly depends on the effective concentration of the elastic chains of the hydrogels and therefore, on the pore size.

**Figure 5:**
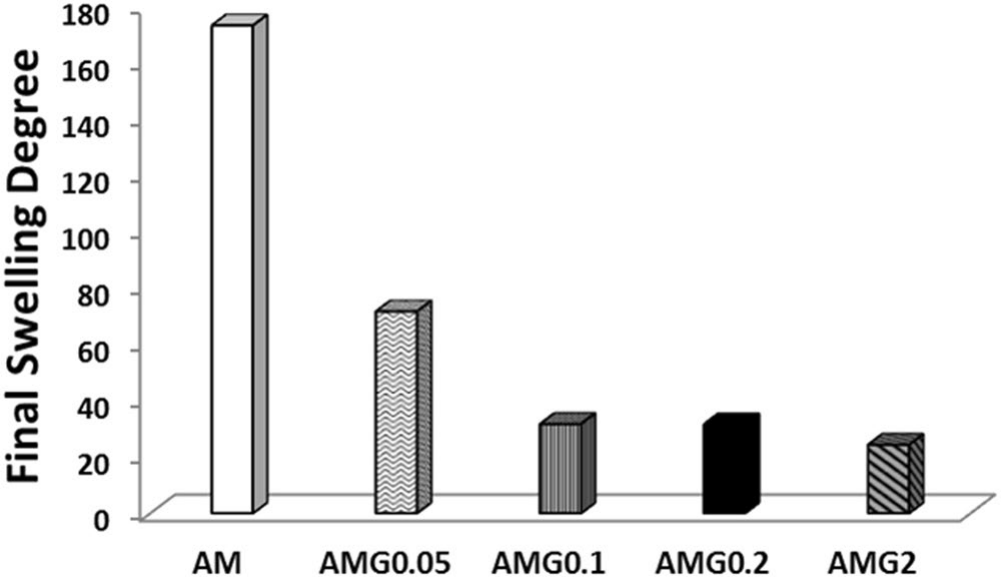
Swelling data of the final materials with several graphene concentrations from 0 mg mL^−1^ (AM) to 2 mg mL^−1^ (AMG2).

Swelling experiments were performed by immersing the freeze-dried materials in doubly deionised water at room temperature (see the experimental details in the Supplementary Information). The final swelling degree decreases when increasing graphene content in the hybrid hydrogels (Fig. 5). The most noticeable changes occur at low graphene quantities where the decrease in swelling degree with increasing graphene loading agrees with the results obtained from the pore size studies, suggesting a main role of graphene in these structures, not just as an embedded nanomaterial but also as a cross-linker. Beyond 0.2 mg mL^−1^ of graphene a physical filling effect can be considered, which would explain the attenuation of the trend in the swelling and pore size studies. More experiments are currently carried out in our labs to better study the behaviour of graphene in these 3D structures.

### Mechanical properties

**Figure 6:**
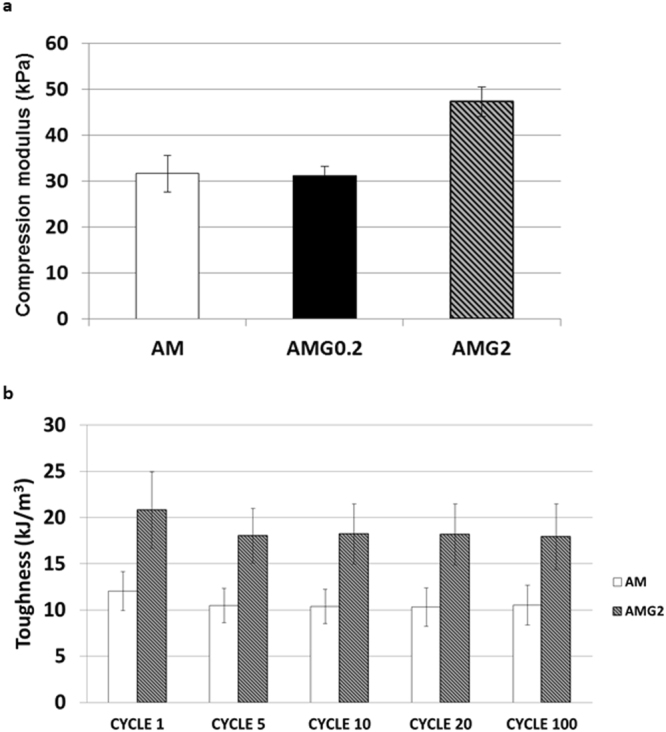
(**a**) Elastic moduli of the AM, AMG0.2 and AMG2 materials. (**b**) Fatigue behaviour of the AM and AMG2 hydrogels.

Mechanical reinforcement is usually one of the main reasons for creating composites based on nanomaterials. Moreover, mechanical properties have been reported by some authors as an important feature to regulate cell behaviour in 3D culture media^47, 48^. Compressive tests were conducted on all the samples. For practical reasons, we only show the compressive Young’s modulus (*E*) for samples AM, AMG0.2 and AMG2, but, although not shown, samples AMG0.05 and AMG0.1 presented identical values in the elastic moduli as AMG0.2. These values are in the range of other materials already used for 3D neuronal growth^49, 50^. The Young’s modulus of the hybrid prepared in the presence of a dispersion of 2 mg mL^−1^ of graphene is considerably higher in comparison with the other two hydrogels. The fatigue behaviour was studied only for AM and AMG2 gels, in order to evaluate if the introduction of graphene could deteriorate the gel and affect the fatigue response. In this case, the compressive toughness of the materials was measured from the area of the stress-strain curve of non-stopped 100 compressive cycles. It is evident that the incorporation of graphene in the hydrogel increases the toughness of the material (Fig. 6(b)). Although a slight loss of toughness of around 13% is observed in both hydrogels during the first five compression cycles, from the fifth cycle onwards, the toughness value remains constant for at least 100 cycles, where no fatigue could be observed. Moreover, the shape of the samples remains intact after these mechanical experiments (Fig. S4 Supplementary Information).

Creep studies were performed on AM, AMG0.2 and AMG2, as stress relaxation has been considered a key feature for the interactions between cells and the extracellular matrices, and also as an important parameter in the design of biomaterials for cell culture^51^.

Creep experiments were conducted at ambient conditions under constant applied load at 15% compressional strain. The results confirm that all the hydrogels represent similar viscoelastic behaviour, as large differences in the stress relaxation profile of the three samples were not observed (Fig. S2, Supplementary Information).

### Neuronal Studies

**Figure 7:**
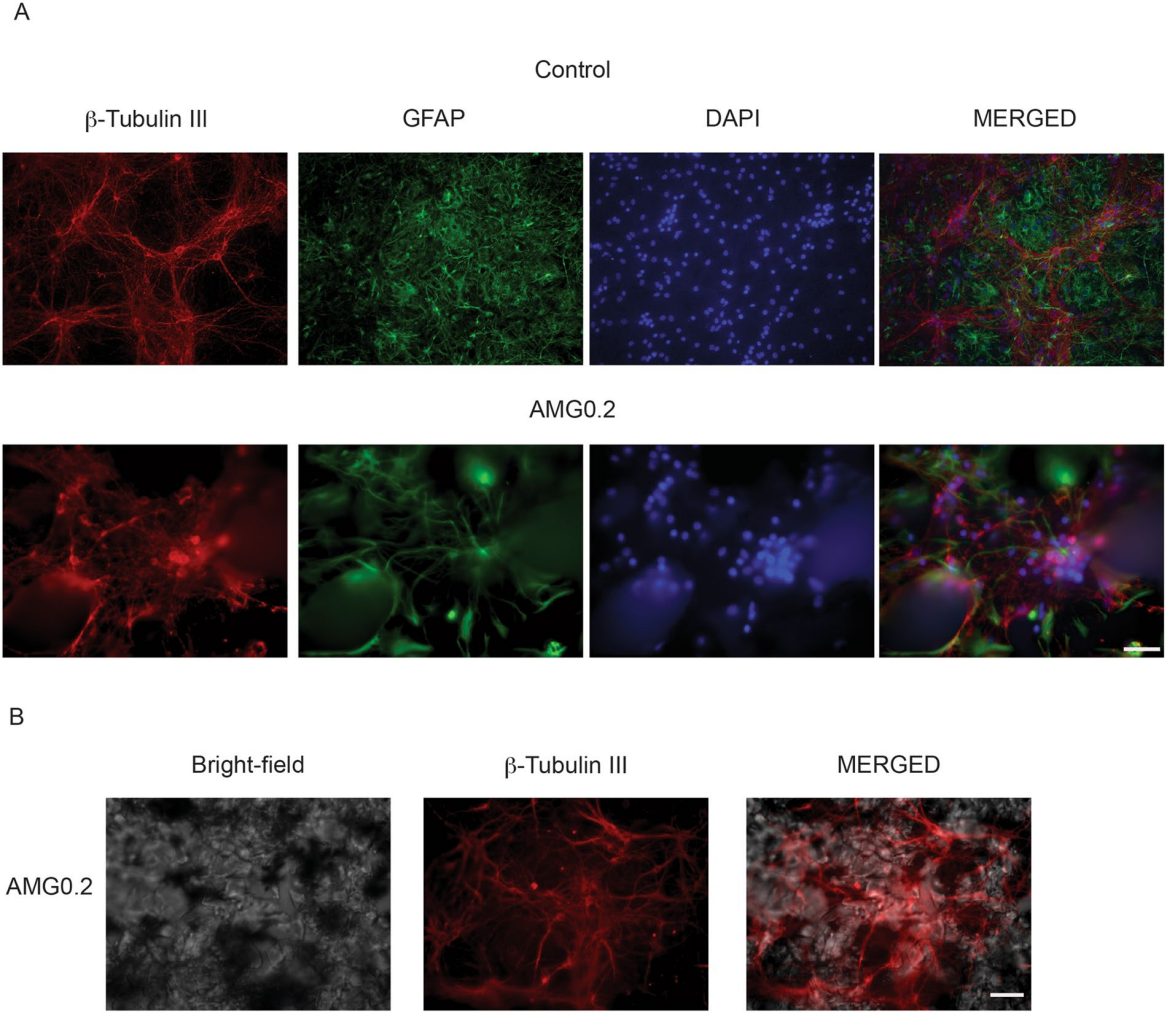
Confocal reconstructions of hippocampal cultures grown (10 DIV) in control (2D- Polyornithine, top) and AMG0.2 hydrogel (bottom). Cells were immunolabelled for β-tubulin III (in red), GFAP (in green) and DAPI (in blue). Scale bar: 50 µm. In the right images, merged channels are shown.

We tested the 3D polyacrylamide scaffold efficacy to support neuronal cell growth and activity and we compared one of the nanocomposite sample (AMG0.2) to its blank AM counterpart. Although different in the pore size ranges, both materials have pores within dimensions known to permit cell survival in culture^4, 52, 53^. To investigate the role of graphene in these hydrogels we decided to use the 0.2 mg mL^−1^ condition that shows a pore size within ranges known to favour the development of 3D geometries in cultured neurons^4^. AMG0.2 gels conserved a certain transparency but contained enough graphene (0.1 wt%) to address its potential interference with neuronal growth. The final content of melamine in this hybrid hydrogel is only 5.4 · 10^−4^ wt%, which is not comparable to the amount of cross-linker used to form the nanocomposite sample (0.1 wt%). The intrinsic role of graphene would be thus revealed, with potential interest in further scalability and applications. We cultured cells in three different conditions: in traditional 2D polyornithine-layered substrate (control)^4^ and in the hydrogels, cross-linked in the presence (AMG0.2) or in absence (AM) of graphene. After 10 DIV we compared neuronal growth by immunofluorescence techniques and confocal microscopy. As shown in Fig. 7, we visualised neurons by immuno-labelling for the specific cytoskeletal component β-tubulin III; astrocytes were identified by the specific cytoskeletal component glial fibrillary acidic protein (GFAP)^4^. Despite repeated trials (3 different culture series) hippocampal cells when seeded on AM hydrogels did not adhere to the structure and, even more, barely grew. We detected only occasionally few clusters of small β-tubulin III-positive cells, virtually deprived of outgrowing axons, as shown in Fig. S3 (Supplementary Information). To note, also astrocytes did not develop under these culturing conditions (Fig. S3). These results indicate an intrinsic low biocompatibility for neurons of the AM material, that does not allow neuronal and glial adhesion, maturation and survival in this artificial structure. Conversely, hydrogels enriched by small amounts of graphene (AMG0.2) allowed for proper neuronal and glial development. Figure 7(A) shows examples of confocal reconstructions of cultured β-tubulin III positive cells (in red) and GFAP positive ones (in green) at 10 DIV in control substrates and in AMG0.2 hydrogels; in both conditions nuclei are visualised by DAPI (in blue). Cells were seeded on top of both substrates and after 10 DIV we observed no differences in their relative Z profile reconstruction (see Experimental Section). In these examples, in 2D control cultures nuclei were distributed within a section corresponding to 18 µm thickness (on average 15 ± 4 µm, n = 13 visual fields, 3 series of cultures). Similarly, when reconstructing the Z profile in AMG0.2 scaffold, the depicted thickness was of 21 µm (on average 18 ± 5 µm, n = 15 visual fields, 3 series of culture)^4^. The zeta-stack reconstruction revealed that hippocampal cells were able to grow within the porous niches present on the surface of the graphene-based hydrogels, but they did not migrate deeper in the structure^4^. This is further sustained by the reconstruction of Fig. 7(B), that shows the bright-field image of the AMG0.2 hydrogel (left) and the β-tubulin III positive-neurons grown on the structure (middle). The superficial distribution of neurons is highlighted by the merged image (left). Regardless the lack of complex 3D geometries, neurons and glial cells apparently developed in AMG0.2.

**Figure 8:**
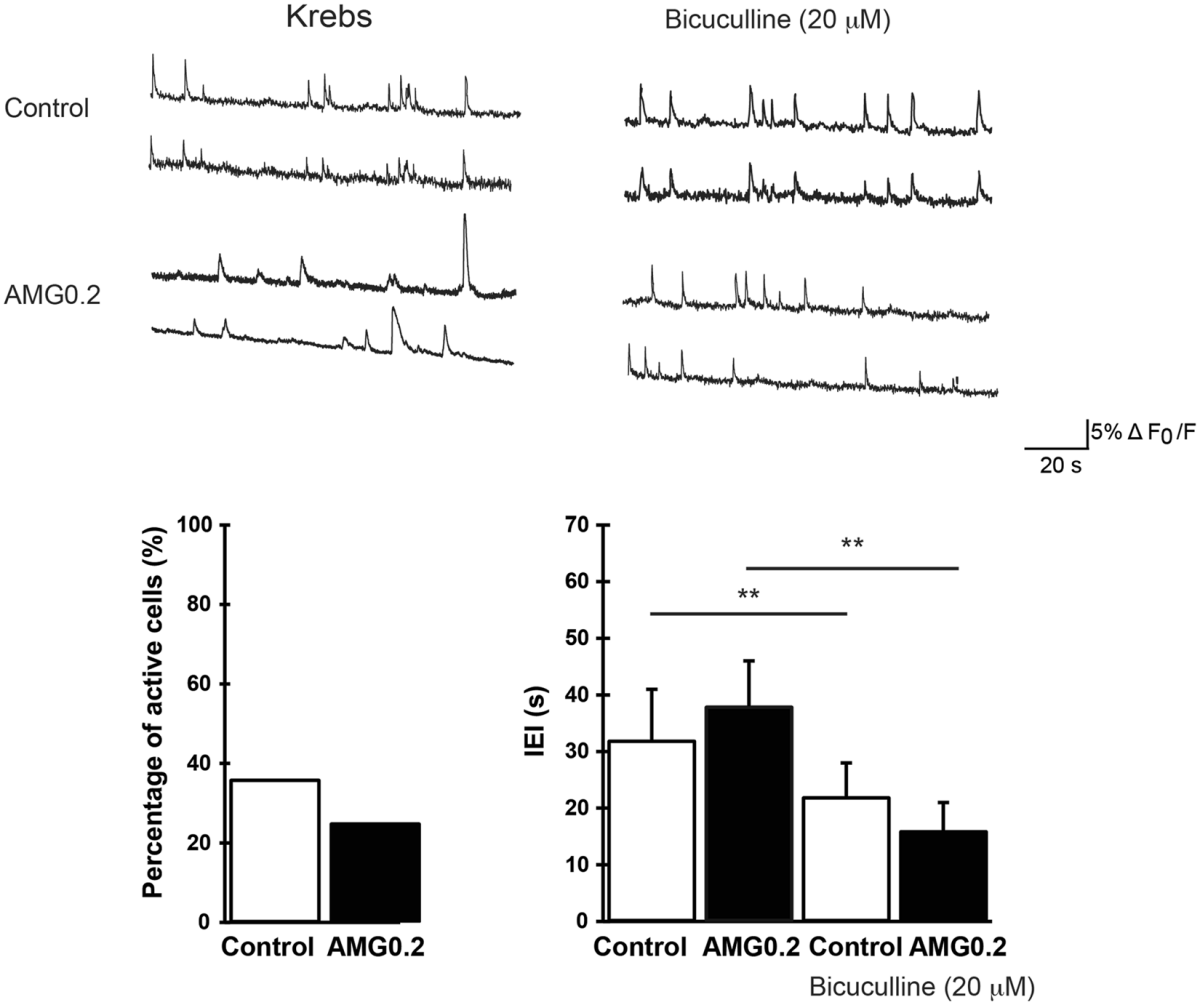
Top tracings) Repetitive Ca^2+^ - events spontaneously (left panel, in standard saline, Krebs) or bicuculline-induced (right panel) recorded in hippocampal cultures at 10 DIV (two neurons were selected from each field). Bottom) Histograms summarise the percentage of spontaneous active cells (left) and the average values of the inter-event interval (IEI, right) in control and AMG0.2 cultures, in standard saline (left columns) and in the presence of bicuculline (^**^P < 0.01, Student’s t-test; data are mean ± SD).

To investigate the presence of neuronal activity emerging from the newly formed networks, we used fluorescent calcium imaging^4^. At 8–10 DIV neurons are usually mature and active and display synaptic signaling spontaneously, traditionally mediated by glutamate and GABA_A_ receptor mediated-synaptic pathways^4, 35, 36^. With our imaging set up we sampled representative regions of 120 × 160 μm^2^. Neurons were stained with the membrane permeable Ca^2+^ dye Oregon green 488 BAPTA-1 and simultaneously visualised within the sampled area. On average, 6 ± 2 fluorescent cells were detected in the recorded field. In Fig. 8, recordings from active fields in control and AMG0.2 substrates are shown (top tracings, two sampled cells for each field). Spontaneous Ca^2+^ activity usually appeared as spontaneous bursts of activity fully blocked by TTX (1 µM) applications (n = 5 fields in control, n = 6 fields in AMG0.2; not shown). TTX is a well-known blocker of voltage-gated fast Na^+^ channels^54^, responsible for the generation of action potentials. In our recordings, spontaneous Ca^2+^ activity was detected in 36% of control cells visualised in each field (21 out of 58 neurons, n = 8 fields) and, similarly, in 25% AMG0.2 cells (16 out of 64 neurons, n = 9 fields, AMG0.2; Fig. 8, left plot). In control cultures, Ca^2+^ oscillations were characterised by inter-event interval (IEI) of 32 ± 7 s (n = 21 cells) that is similar to that measured in AMG0.2 cultures (38 ± 5 s, n = 16 cells; right plot in Fig. 8).

In a second set of experiments we pharmacologically blocked GABA_A_ receptors by bicuculline (20 µM, 15 min) application. This antagonist of synaptic inhibitory components is known to potentiate rhythmic activity patterns^4, 55, 56^. In fact, in both groups, in respect to spontaneous activity, the addition of bicuculline significantly decreased the IEI average values (P < 0.01, Student’s t-test)^4^. In Fig. 8, fluorescence tracings show the appearance of Ca^2+^ episodes brought about by bicuculline in active cells that, on average, display IEI of 22 ± 4 s in control (n = 21 cells) and, similarly, of 16 ± 6 s in AMG0.2 cultures (n = 16 cells; Fig. 8, right plot). These experiments confirm that the neuronal signaling detected by calcium imaging is entirely due to neurons firing action potentials and communicating via GABA_A_ and glutamate receptor-mediated synaptic pathways^4^.

The main result obtained from our neurobiological studies is that the presence of graphene inside the structure of the hydrogel is crucial for allowing cell adhesion and the formation of mature and active synaptic networks. In fact, only sporadic clusters of few neurons can be found in the blank hydrogel, in the absence of proper axon formation. Ultimately, neurons only grew in the scaffold synthesised by using a graphene dispersion in water with a concentration of just 0.2 mg mL^−1^, but not in the absence of the nanomaterial. The fact that the hybrid AMG0.2 displays similar viscoelastic behaviour, Young’s modulus and compressive toughness as AM suggests that the differences in cell attachment might be related to other mechanisms, such as the adsorption levels of adhesion proteins^57^: proteins could be more effectively adsorbed on the substrate in the presence of graphene. In addition, the size and the shape of the pores are determinant parameters for dictating 3D geometries of neuronal growth and thus activity^4, 58^. As already discussed, the pore size of this kind of scaffolds decreases in the presence of graphene. It is known that the pore size can crucially govern the geometry of 3D neuronal networks but does not determine cell survival. In fact, previous works have shown hippocampal neurons growing on different 3D scaffolds, despite the large differences in pore sizes^4, 52, 53^. Certainly, the mechanisms responsible for the graphene ability to improve cell adhesion need to be fully understood and deserve further investigation. One possibility is that the graphene-driven cross-linking affects the porous surface topography directly favouring neuronal migration within the scaffold, axonal elongation, differentiation and growth. Alternatively, other, yet unexplored, graphene features might promote the deposition of the extracellular matrix (ECM), therefore indirectly affecting neuronal growth.

The neuronal networks grown in control and AMG0.2 display basically similar Z projection (≈20 µm thickness), thus in the graphene-hybrid hydrogels, neurons did not develop in a genuine 3D geometry^4^. This is further suggested by the observed similarity in the activity patterns when recorded by calcium imaging in the two culturing conditions^4^. However, in both networks, episodes of calcium activity, whose neuronal and synaptic nature was supported by TTX experiments, were reflecting spontaneous synaptic activity. Based on these results, we are carrying out further experiments to adjust the pore size dimensions and interconnectivity in the graphene-based hydrogels in order to improve the growth and migration of neurons deeper within the sample, favouring by this way 3D neuronal network connections.

## Conclusions

We have designed hybrid graphene hydrogels as new 3D scaffolds for neuronal growth. Acrylamide was used as main monomer for the formation of the polymer networks and the radical polymerization was conducted in aqueous graphene dispersions of different concentrations. The scaffolds were thoroughly characterised, and the compressive moduli were measured, being higher for the sample that contains a noticeable amount of graphene. Moreover, no significant fatigue behaviour was observed in the studied samples, even in the hydrogel with the highest amount of graphene. The most important result observed from the neuronal studies is that neuronal networks are only visualised in the hybrid graphene hydrogel, but not in the scaffold without graphene. The mechanical properties of the scaffold do not seem to be critical for neuronal growth in the presence of graphene and thus an intrinsic beneficial role of graphene is revealed.

## Electronic supplementary material

Supplementary info
