# Supplementary material for: Graphene Improves the Biocompatibility of Polyacrylamide Hydrogels: 3D Polymeric Scaffolds for Neuronal Growth
Source: Sci Rep. 2017 Sep 8;7:10942. doi: 10.1038/s41598-017-11359-x (PMC5591295; doi:10.1038/s41598-017-11359-x)
Supplement: Supplementary file 1 — Supplementary info [file 41598_2017_11359_MOESM1_ESM.pdf]

Supplementary Information

Graphene Improves the Biocompatibility of Polyacrylamide Hydrogels: 3D Polymeric Scaffolds for Neuronal Growth

Cristina Martín[1, 2], Sonia Merino[1], Jose M. González-Domínguez[1], Rossana Rauti[3], Laura Ballerini[*,3], Maurizio Prato [*,2, 4, 5] & Ester Vázquez[*,1]

[1]Organic Chemistry area, Faculty of Chemical Science and Technology-IRICA, University of Castilla-La Mancha, Avda. Camilo José Cela 10, 13071, Ciudad Real, Spain.

[2]Department of Chemical and Pharmaceutical Sciences, University of Trieste, Piazzale Europa 1, 34127 Trieste, Italy.

[3]International School for Advanced Studies (SISSA), via Bonomea 265, 34136 Trieste, Italy.

[4]Carbon Nanobiotechnology Laboratory, CIC biomaGUNE, Paseo de Miramón 182, 20009 Donostia-San Sebastián, Spain.

[5]Ikerbasque, Basque Foundation for Science, E-48011 Bilbao, Spain.

Correspondence and requests for materials should be addressed to L.B. (email: laura.ballerini@sissa.it), M.P. (email: prato@units.it) or E.V. (email: ester.vazquez@uclm.es)

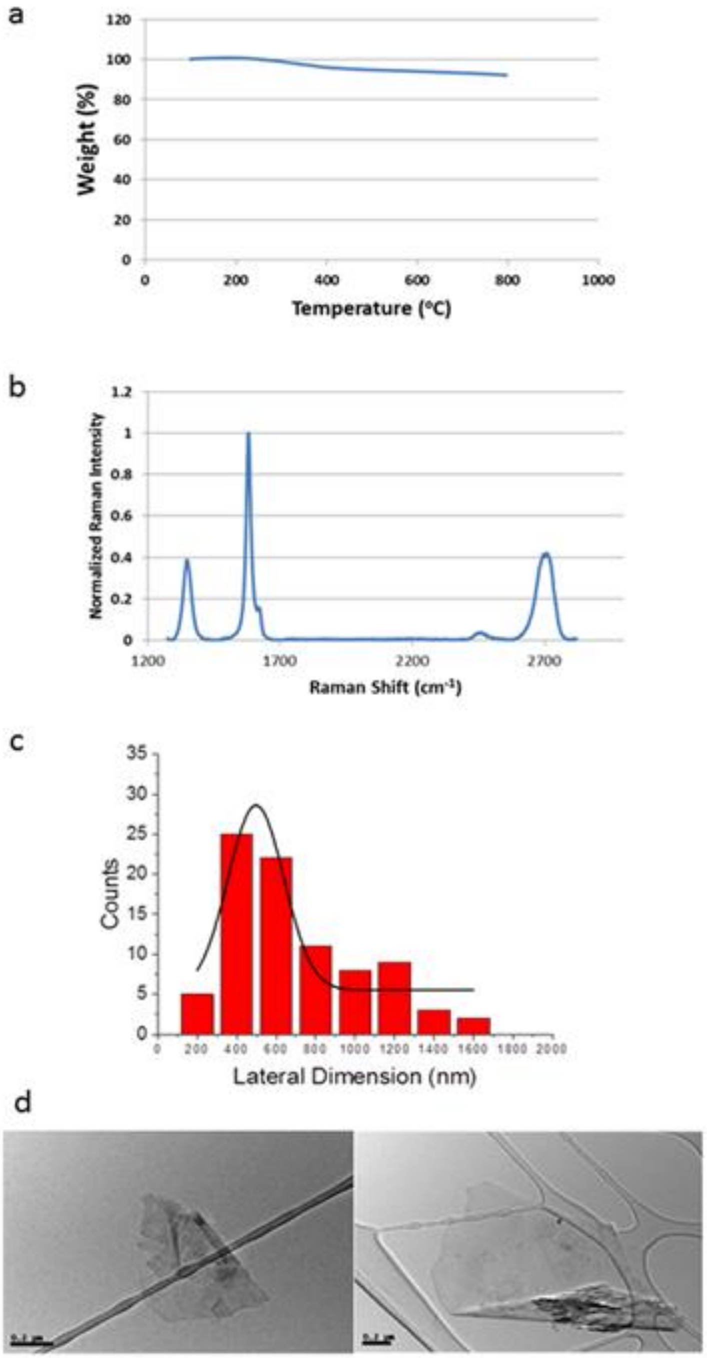

Supplementary Figure S1. Characterisation of the not oxidized graphene used to create the hybrid hydrogels: a) thermogravimetric analysis (repeated three times), b) normalised Raman spectrum, c) distribution of the lateral dimension of the graphene sheets from transmission electron microscopy (TEM) images and d) representative TEM images.

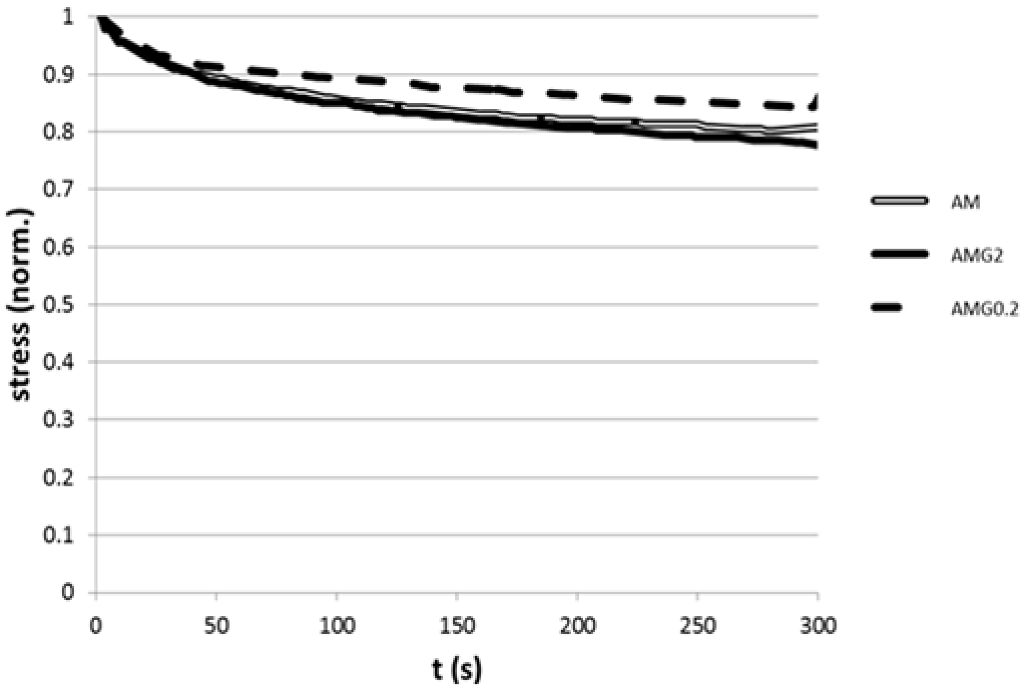

Supplementary Figure S2. Stress relaxation test on AM, AMG2 and AMG0.2 (15% compressional strain).

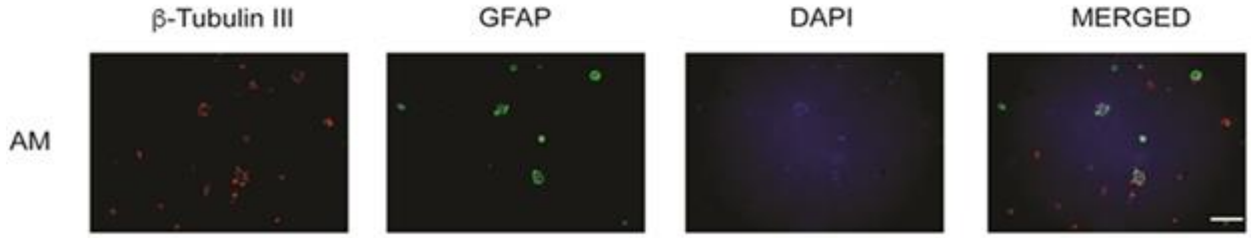

Supplementary Figure S3. Confocal reconstructions are shown to visualize neurons and glial cells grown on AM hydrogel (β-tubulin III, in red; GFAP, in green; DAPI in blue; right image shows merged channels). Scale bar: 50 μm.

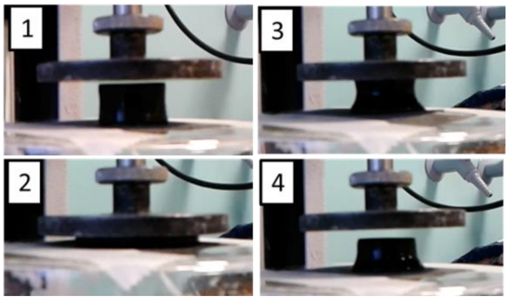

Supplementary Figure S4. Representative pictures of AMG0.2 in the just prepared swelling degree during mechanical testing: 1,2 and 3): during fatigue experiments, 4): after 100 compression cycles.

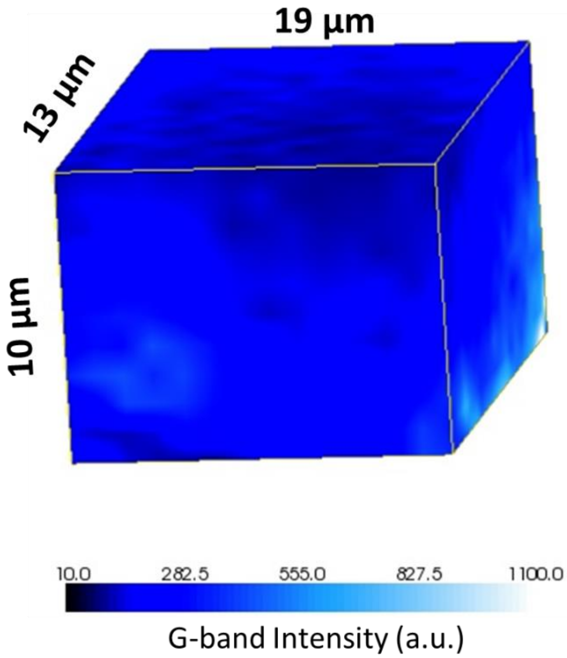

Supplementary Figure S5. 3D map of the G-band intensity in AMG2. According to the dimensions of the probed volume (10×13×19 µm) and given that we registered 3080 spectra during the mapping, we end up with a pixel size of 0.8 µm^3.

Kind of graphene	Methodology	I(D)/I(G)	Reference
**Graphite exfoliated with surfactant (SDBS)**	Mechanical / Liquid phase (aqueous)	~0.35	J. Am. Chem. Soc. 2009, 131, 3611
**Graphene oxide (Improved Hummers)**	Chemical	~ 0.75	ACS Nano 2010, 4, 4806
**Reduced graphene oxide (L-lysine as reducing agent)**	Chemical	1.11	J. Mater. Chem. A 2013, 1, 2192
**Graphite exfoliated with 1-pyrenesulfonic acid**	Mechanical / Liquid phase (aqueous)	~0.6	Carbon 2013, 53, 357
**Graphite exfoliated with alkyl compounds**	Mechanical / Liquid phase (organic)	1 – 1.8	J. Phys. Chem. Lett. 2016, 7, 2714
**This work**	Mechanochemical (aqueous)	~0.4	Nanoscale 2016, 8, 14548

Supplementary Table S1. Brief comparison among some graphene materials produced by mechanical or chemical methods.

Swelling Behaviour. Experimental Details.

Swelling studies were carried out in lyophilised hydrogel samples after washing and bringing them to the maximum expansion degree. These samples were immersed in ultrapure Milli-Q water (10 mL) at room temperature. All the samples have a similar volume of approximately 0.5 cm^3. The weights of the swollen hydrogels were recorded at regular time intervals until they reached a constant weight. Water excess on the surface of the gels was removed with a filter paper before weighing. The swelling degree was then calculated according to the following Equation (1):

$$\text{Swelling Degree} = \frac{W_t - W_0}{W_0} \qquad (1)$$

where W_t is the weight of the gels at time *t* and W_0 is the weight of the dry gels at time 0. The swelling degree was also defined as the final swelling equilibrium after one day, when the weight of the samples was found to be constant.
